# Tomographic Particle Image Velocimetry using Smartphones and Colored Shadows

**DOI:** 10.1038/s41598-017-03722-9

**Published:** 2017-06-16

**Authors:** Andres A. Aguirre-Pablo, Meshal K. Alarfaj, Er Qiang Li, J. F. Hernández-Sánchez, Sigurdur T. Thoroddsen

**Affiliations:** Division of Physical Sciences and Engineering, King Abdullah University of Science and Technology (KAUST), Thuwal, 23955-6900 Saudi Arabia

## Abstract

We demonstrate the viability of using four low-cost smartphone cameras to perform Tomographic PIV. We use colored shadows to imprint two or three different time-steps on the same image. The back-lighting is accomplished with three sets of differently-colored pulsed LEDs. Each set of Red, Green & Blue LEDs is shone on a diffuser screen facing each of the cameras. We thereby record the RGB-colored shadows of opaque suspended particles, rather than the conventionally used scattered light. We subsequently separate the RGB color channels, to represent the separate times, with preprocessing to minimize noise and cross-talk. We use commercially available Tomo-PIV software for the calibration, 3-D particle reconstruction and particle-field correlations, to obtain all three velocity components in a volume. Acceleration estimations can be done thanks to the triple pulse illumination. Our test flow is a vortex ring produced by forcing flow through a circular orifice, using a flexible membrane, which is driven by a pressurized air pulse. Our system is compared to a commercial stereoscopic PIV system for error estimations. We believe this proof of concept experiment will make this technique available for education, industry and scientists for a fraction of the hardware cost needed for traditional Tomo-PIV.

## Introduction

Flow visualization and quantitative velocity measurements are the foundations of experimental fluid mechanics. They have, with the other pillars of theory and numerical simulations, built up our understanding of the dynamics of complex or turbulent flows. Since the invention of the CCD sensor the advances in digital cameras and computational power have gone hand-in-hand as their electronic fabrication methods have taken a parallel track. This is particularly true in the recent advances in smartphone technology, where a camera and a computational brain have been integrated into a rapidly developing combined device and the economics of scale have allowed the addition of advanced features into these mass-produced camera-sensors, at minimal cost. Indeed as smartphones have become ubiquitous in the general population, they are equipped with high quality sensors such as gyroscopes, accelerometers, GPS and one or two CMOS camera sensors. These features allow people to communicate, record vast amounts of information and even monitor their health at a fraction of the cost of similar commercial scientific cameras and other sensors used in industry and research, thereby, becoming a valuable tool in everyone’s daily life. Herein we demonstrate that the recent very high pixel-count of a few of these camera sensors can be combined together for measuring three-dimensional velocity fields.

Particle Image Velocimetry (PIV) is the most powerful modern technique to measure extended velocity fields^1–4^. This relies on seeding the flow-volume with small tracer particles which are illuminated with pulsed light and digital cameras capture images of their displacement with time. The velocity field is subsequently extracted through image correlations with specialized software. The power of this technique has grown directly with the increased capabilities of the available digital cameras^3^, both in terms of total number of pixels as well as the available frame-rates, for improved space and time resolution. Specialized PIV systems were initially based on dual-frame cameras, but are now being replaced with high-speed video cameras, which allow time-resolved flow evolution even at high Reynolds numbers. Increased sensor capabilities and by combining more than one camera, have led to the development of stereoscopic PIV^4^, where two cameras at different angles allow the determination of both the in- and out-of-plane components of the velocity in a single plane (2D-3C). Scanning the laser plane has allowed full 3-D measurements^5^, but only for slow motions, where the flow does not change appreciably during the scanning time. The most recent evolution of this technique, is Tomographic PIV where the instantaneous three-component velocity field is determined in a volume-slice^6^. Here three or more synchronized cameras view the flow from different directions and record the particles illuminated with a pulsed laser volume-slice. Including a high-precision calibration, the frames from all the cameras are used in an iterative algorithm using Multiplicative Algebraic Reconstruction Technique (MART) based on a Multiple Line of Sight (MLOS) initialization^7^. This allows 3-D reconstruction of the particle locations in the volume to obtain three-component velocity fields (3D-3C), based on multi-step 3-D cross-correlation^6^. However, multiple research-level cameras, a high-power laser and a synchronization unit are an expensive proposition for most labs and educational institutions. Lasers also pose safety concerns, especially in an educational setting. Herein, we address both the cost and the illumination technique, using the smartphone cameras and LED lighting. Other researchers have recently focused on reducing the cost of the hardware. For instance, single shutter commercial cameras have been used in an effort to reduce costs by Discetti *et al*.^8^. Moreover, high power LEDs have been used as an illumination source, replacing the expensive laser systems^9–13^. Buchmann *et al*.^14^ have been able to reconstruct grid turbulence in 3D using a system of multiple high-sensitivity CCD cameras and LEDs as light sources. They observe forward scattered light from the particles, capturing pairs of images with Δt = 5 ms and exposure time of 150 μs.

Casey *et al*.^15^ have improved the depth of the measurements volume of Tomo-PIV, by rapidly scanning a volumetric laser-slice, rather than a laser plane. This can resolve much larger number of particles in the depth direction, to produce more vectors in the flow-volume.

Alternatives to Tomo-PIV are based on 3-D particle tracking^16, 17^, or triple aperture imaging^18^. Color has here been used to simplify the data reduction and reduce ambiguity in the reconstruction with a single camera^12, 19, 20^.Watamura *et al*.^21^ have used space color-coded light to extract the 3-D location of particles within a volumetric illumination produced with an LCD projector, using a single camera and thereby reducing costs significantly.

A combination of monochromatic LEDs, shadows and 3D reconstruction techniques has been reported by Klinner and Willert^22^. In this investigation they used the droplets from a cone spray with the goal of reconstructing their spatial distribution in three dimensions.

Similarly, Particle Shadow Velocimetry (PSV) has proven to be an effective alternative to PIV that uses light scattered from seeding particles^23^. This technique registers the shadows of the particles instead. Thus, the light intensity required to detect the location of the particles is reduced. Estevadeordal and Goss^23^ have reported the use of color coded LED flashes to tag different instants, consequently computing the 2D velocity fields from single images, as we do herein. Additionally, thorough studies have been carried out by McPhail *et al*.^24^ to estimate and correct for color distortions in PSV due to different refractive indexes for different wavelength in the optical path, especially when the light passes through the plexiglass container walls.

Taking advantage of the capabilities of smartphones, 2D PIV has already been implemented to observe a water jet axially cut by a CW laser light sheet by Cierpka *et al*.^25^. In these experiments video at 240 fps and a resolution of 1280 × 720 pixels is recorded with smartphones, reducing greatly the hardware cost for this kind of system.

In this report we show that low-cost smartphone cameras and LED illumination can be used to perform Tomo-PIV based on colored shadows (Tomo-PSV). We demonstrate this by using single frames from 4 smartphone cameras, while using colored shadows to imprint two or three images of opaque particles seeded within a vortex ring. We thereby combine previous distinct techniques such as PSV, using LED illumination and smartphone camera sensors in a novel tomographic setup, thereby reducing the cost of the hardware.

## Experimental Method

### Overall Setup

**Figure 1:**
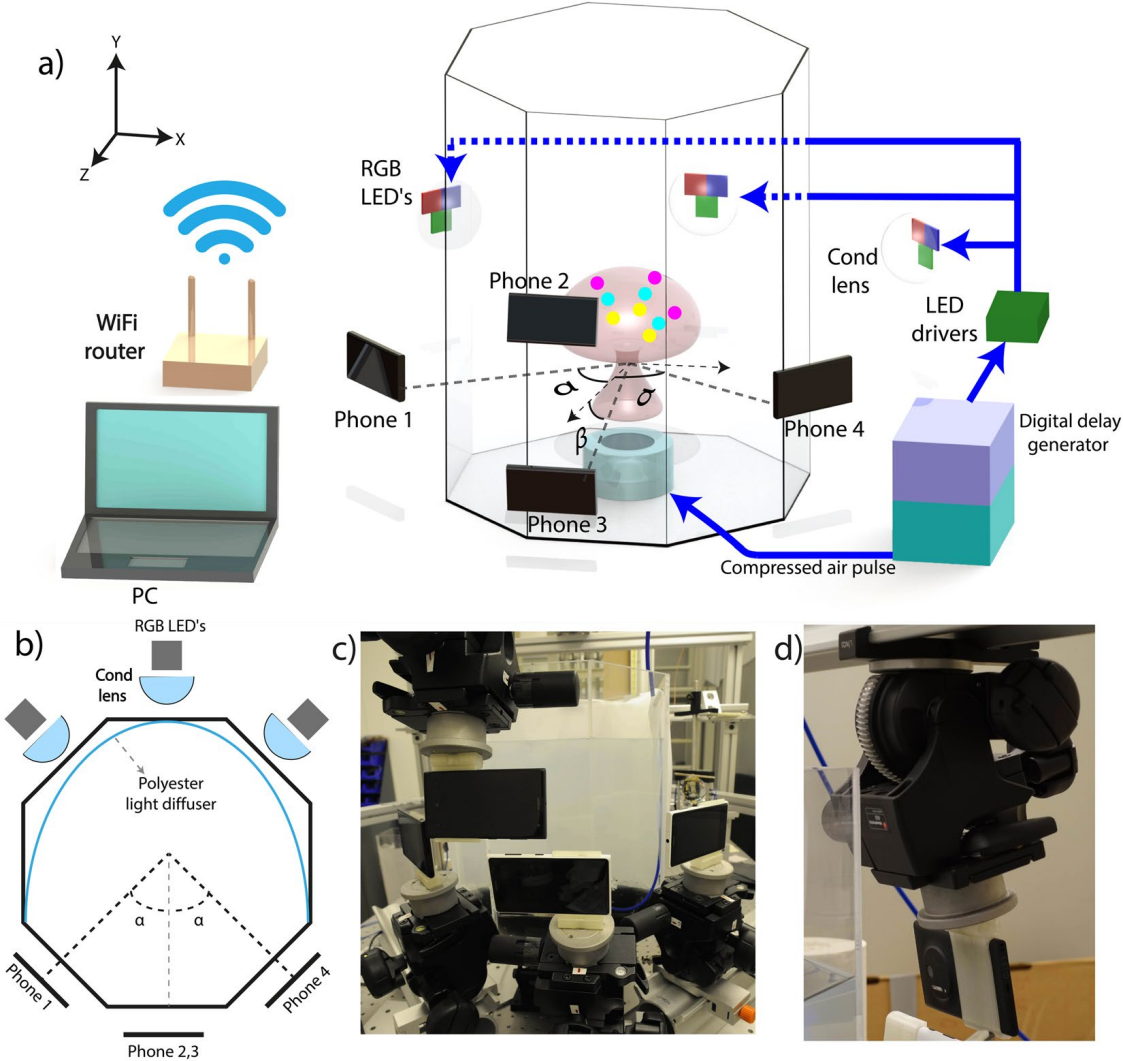
(**a**) Schematic experimental setup of the smartphones, vortex ring generator, LEDs and the components required to trigger and synchronize the system, while capturing three different time steps shown as yellow, cyan and magenta colors. The angle between the cameras are α = 45° and β = 10°. (**b**) Top view of the setup. (**c**) Actual photography of the setup, showing the mounting structure for precise and fine adjustment of the smartphones. (**d**) Detail of the support structure and 3d printed holder.

Figure 1 shows the overall setup of the flow and optical systems. On a first iteration, we generate a vortex ring translating vertically up inside an octagonal tank filled with pure water. On a second iteration the water was replaced by an aqueous solution of sodium chloride, with a density (ρ) of 1.18 g/cm^3^. This aqueous solution is used instead of pure water due to the limited availability of tracer particles. In this way, the difference in density does not affect as much the quantitative results, as discussed further in a later section. The ring is seeded with black opaque particles. Four smartphone cameras are arranged to observe the particle motions from different angles. The cameras are triggered simultaneously with a long exposure to observe the flow in a darkened room. The three sets of LED flashes are synchronized, so all cameras record each colored shadow projected by the opaque particles at the same instant. This is done for each of the three colors to record the three instants onto the same image frame. A summary of the system components and specifications is presented in Table 1.

**Table 1:** Summary of parameters used for capturing tomographic PIV images of a vortex ring.

Flow	Vortex ring	Generated by pulsed membrane
	pulse width	150 ms
	air pressure	2.3, 3.2 bar
LED lighting	Model	RGB PT-120 Luminus
	Pulse width	80 μs
	Δt	1300, 1000, 500 μs
	Current	15 A
	Voltage	12 V
Camera properties	4x Nokia Lumia 1020	Back camera
	Type	CMOS
	Lens	26 mm
	Aperture	F# 2.2
	Sensor size	2/3″
	Resolution	38 Mpx
	Pixel pitch in sensor	1.12 μm
	Color depth	8 bit
Seeding particles	Manufacturer	Cospheric
	Type	Black polyethylene
	Density	1.28 g/cm^3^
	Size	212–250 μm
Image setup	Viewing angles	±45°, ±10 °
	Total PIV system angle	90°
	Particle diameter	6 px
	Pixel size in focal plane	38 μm/px
	Particle density N	0.005 ppp
	Source Density (N_s_)	0.3

### Cameras and Imaging System

Due to their especially large pixel-count, four Nokia Lumia 1020 smartphone cameras are selected to observe the volume of interest from four different angles. The main camera has a 41 Mpx sensor (effectively 38 Mpx) and has a 2/3 inch sensor size, which is quite large for smartphone standards. The pixel pitch-size is 1.12 μm with a Bayer filter, providing 8-bit color depth per channel, i.e. 256 intensity levels for each color. The lens system has a fixed aperture of f# 2.2 and fixed focal length of 26 mm^26^. The images are saved automatically in DNG (RAW) format, before the default commercially supplied color-interpolations of the pixel-arrays are performed. These characteristics allow a depth of field in the experiment of approximately 100 mm where the particles are observed in reasonable focus. The small pixel size in the sensor compromises the sensitivity to illumination. However, this is overcome by using high-power LEDs as a light source and registering shadows rather than the scattered light or fluorescence from particles, typically employed in PIV. This decreases significantly the illumination intensity requirements.

The control settings of the four cameras are synchronized using simultaneous html commands transmitted through a local wireless network from a PC. These commands set the exposure time, white balance, gain, ISO and then control the triggering time. The ISO is set to 200 minimizing noise in the sensor. The exposure time of all the smartphones is set to 1 second, such that all the cameras can sense the light coming from the LEDs at the same time, overcoming any delay in the trigger of each camera due to the network ping times.

In order to minimize any misalignment or movement of the individual cameras, a stiff customized 3D-printed holder made of polylactic acid (PLA) was fabricated in-house. This holder enabled a firm attachment of the smartphones to a geared tripod-head for fine positioning and angle adjustments. The geometry and the position of the cameras are illustrated in Fig. 1(a,b). The cameras view the flow through 3 adjacent windows of the octagonal tank. Two cameras look through the central window, at β = ±10° from the horizontal, while the other two look horizontally through the adjacent windows, at α = ±45° from the central window. Here we have not used fluid-filled prisms to minimize the distortions of light-rays going through the air-plexiglass interface^15^. Such setup could improve results in future work.

### Vortex ring generator

**Figure 2:**
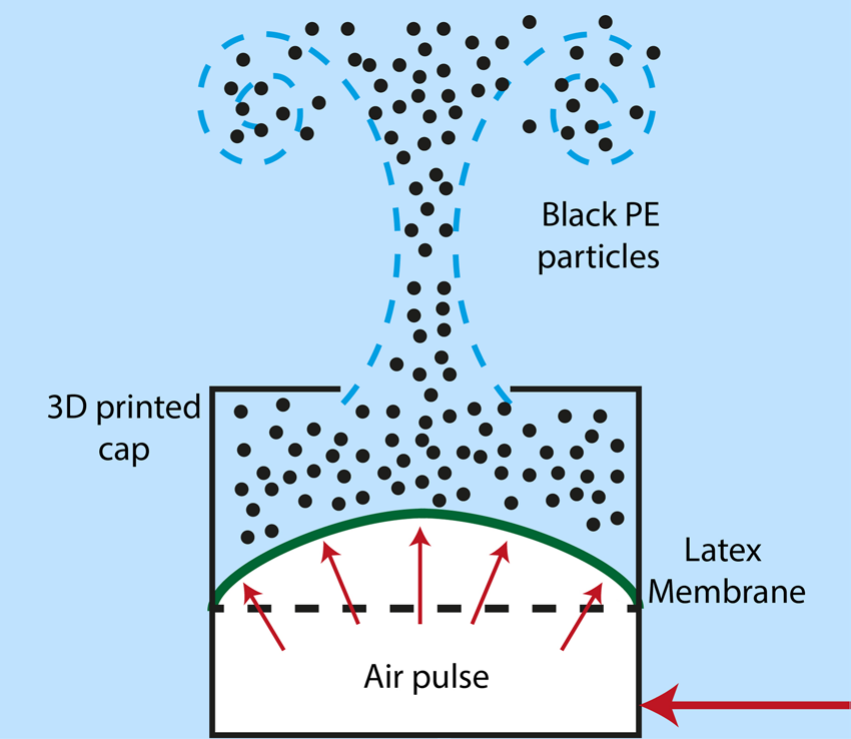
Schematic drawing of the vortex ring generator. Pulsed air is introduced into the enclosed bottom chamber, pushing a latex membrane to force out a fraction of the fluid in the chamber through a circular orifice, thus creating a vortex ring. The liquid inside the chamber has been pre-seeded with black micro-spheres.

In order to test our technique, a vortex ring is produced and observed as it travels up inside an octagonal water tank, through the field of view of the four cameras. Figure 2 shows the device used to generate this vortex ring. It is placed at the bottom of the 390 mm deep plexiglass tank with a horizontal width of 340 mm. Each of the octagonal flat segments is 145 mm wide. Figure 1(a) shows the orientation of the coordinate system we use. This device consists of a circular chamber of 100 mm in diameter, which is covered with an impermeable, elastic latex membrane that isolates both sides of a cylindrical container. The bottom side of this container is sealed from the water and it is connected by a hose to an air flow controller that is operated in pulsed mode. The flexible membrane is enclosed at the top by a 3D printed cap with a smaller circular centered hole of 60 mm in diameter. The duration and pressure level of the air pulse, delivered into the chamber, can be regulated and is synchronized with the LEDs via a digital delay generator (DG645 from Stanford Research). The air pulse drives the membrane upwards pressurizing the chamber and forcing the water to pass through the reduced cross section in the cap, accelerating the flow and finally separating the boundary layer to generate the vortex ring, as explained in Fig. 2. As expected, increasing the pressure of the air pulse gives more impulse to the membrane and produces a faster translation and rotation of the vortex ring. We selected two pressure levels, of 2.3 and 3.2 bar, to drive vortices at different translational velocities of 0.15 to 0.25 m/s, which required time steps between flashes of Δt = 1000 and 500 μs, for the case of pure water, and Δt = 1300 μs when using the sodium chloride solution.

### Flow tracers

The material density of the particles in traditional PIV systems should be nearly neutrally buoyant to follow the fluid motion. Simultaneously, opaque particles are necessary to block the light from the camera sensors and obtain sharper shadows in the recorded images. Due to the color nature of the illumination technique, the Bayer Filter pattern (that uses an array of 2 × 2 GR/BG pixels) present in most color sensors and the fixed non-interchangeable optics of the smartphone cameras, present a challenge to accurately locate the tracer particles. Therefore a particle size of at least 4 to 5 pixels (>200 μm in the actual configuration) is necessary to minimize location bias and diffractive effects. This limited our options in selecting commercially available opaque microparticles ∼250 μm in diameter to seed the flow. Thus, we were forced to use black polyethylene microspheres (Cospheric) with a size range of 212–250 μm and density 1.28 g/cm^3^, as they are opaque and are approximately 6 pixels in the captured images. To reduce the density differences between the particles and the fluid, we use a sodium chloride aqueous solution as the working fluid, to make the particles follow better the fluid motions. For practical reasons the density of this solution is set to ρ = 1.18 g/cm^3^, which is marginally lower than the density of the particles. This allows slow particle sedimentation between experiments without significantly affecting their flow-tracing properties. This reduces the number of defocused particles that add noise in the captured images.

The particles are stirred in the chamber within the cylinder on top of the latex membrane, allowing them to be captured by the vortex ring, tracing the flow field once the air is pulsed. In this way, out-of-focus particles can be minimized, improving the image quality for our demonstration. A schematic of this procedure is illustrated in Fig. 2.

### Illumination

Each camera faces a separate set of multiple colored red, green and blue high power LEDs (Luminus PT-120), see Fig. 1. The LEDs are driven with Phlatlight DKM-136 development kit Drivers, which can be operated in continuous wave or pulsed mode. They are energized by a power supply of 12 V and 15 A each, providing enough power to operate the LEDs at high brightness while protecting them from being overloaded. Each set of RGB LEDs is placed behind an aspheric condenser lens ($\varnothing$ = 75 mm and F = 60 mm, NA = 0.61), concentrating the light in the region of interest. Then, the light passes through a curved diffuser made of a white polyester sheet, distributing the light more uniformly throughout the camera view, as illustrated in Fig. 1(b).

The duration of the illumination faces the common trade-off between sufficient intensity and the need to freeze the instantaneous location of the particles. In our setup we found, via trial and error, that 80 μs flash pulses provide the optimum exposure to both allow sufficient light into the camera sensors, to produce good quality images, while avoiding the smearing of fast moving particles. The pulses are synchronized and activated by a digital delay generator. The time delays between the three different LED color pulses was adjusted for the strength of the vortex rings, i.e. either Δt = 1300, 1000 or 500 μs, thereby capturing three time steps in a single frame. We highlight that much smaller time steps can be achieved by the lighting and camera system, being only constrained by the velocity of the flow and the vortex generation system. The time-response of the LED is less than 200 ns, as reported by Buchmann *et al*.^14^.

Using multi-color illumination can cause systematic bias in the position of the particles recorded in the different colors, due to differences in the diffraction through the transparent walls of the tank. The amount of this diffraction varies slightly with the wavelength of the light. Detailed study of this effect has been carried out by McPhail *et al*.^24^. Further discussion about this bias error will be addressed in a later section.

### Tomographic PIV calibration

**Figure 3:**
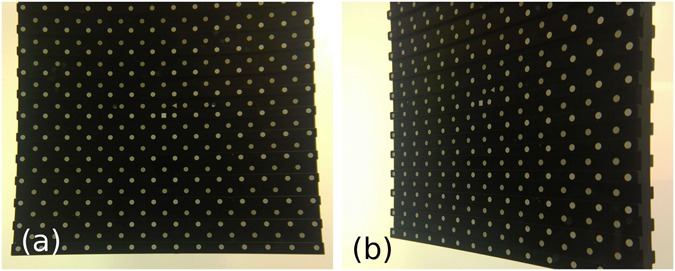
Image of the calibration target (type 22 from LaVision) viewed from two angles. (**a**) From central-bottom camera 3 and (**b**) from the off-axis camera 4. The image shows the optical distortions produced by the lens and by the angled view through the Plexiglas walls.

In order to reconstruct the 3D position of the multitude of tracer particles, it is crucial to obtain a high-quality calibration from *in-situ* images from all four cameras. For this purpose, we use a typical dotted bi-planar calibration target shown in Fig. 3. Here, we used the 30 cm wide bi-planar target (Type 22 from LaVision) which covers a bigger area than the vortex ring. This target is traversed through the volume of interest, precisely controlled with a stepper motor and a translation stage (VT-80 from miCos) over a distance ranging from z = −40 mm to +40 mm in 5 mm steps. This calibration intends to correct any distortion arising from the imperfect lenses and diffraction due to non-perpendicular viewing angles through the tank walls. Significant pincushion distortions are visible at the edges of the calibration target in Fig. 3(a). This original calibration is subsequently improved with the self-calibration algorithm described in a later section^27^.

### Two and Three-Colored Shadows

**Figure 4:**
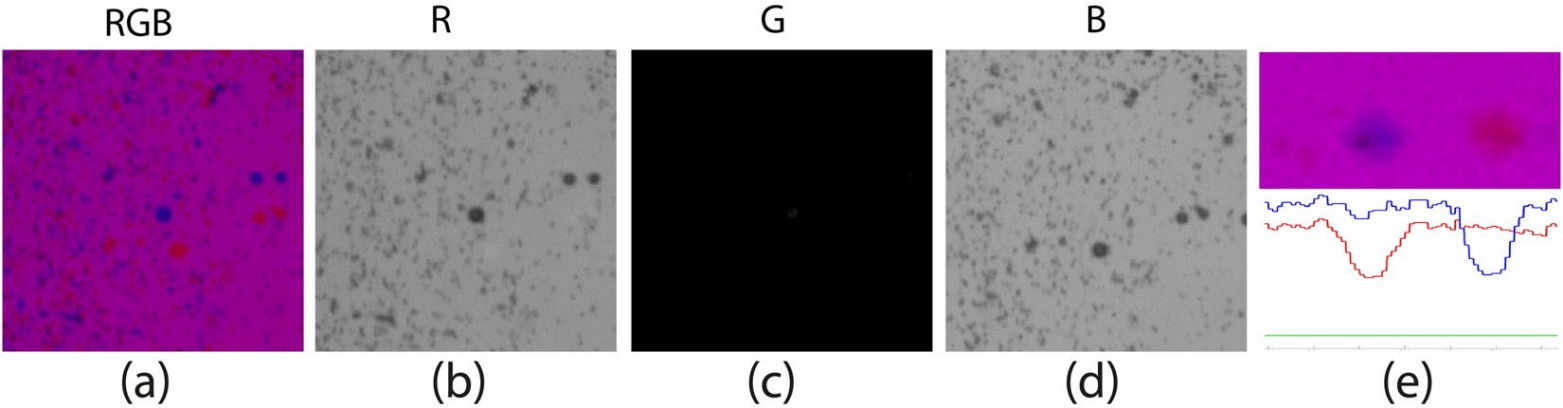
(**a**) Original image using only Red and Blue flashes (obtained with a Nikon sensor). The captured image is separated in its RGB channels red (**b**), green (**c**), and blue (**d**). (**e**) The color intensities cut through the blue and red dots.

Once the cameras are triggered and the vortex ring is generated, the two or three color-coded lights are flashed in a sequence to produce shadows of the particles at different times, t_1_ and t_2_ as well as t_3_. We obtained a pair or triplet of images of each particle in a single frame, as shown in Fig. 4(a). Note that the order of the colors of the dots is reversed from that of the illumination, i.e. if the first flash at t_1_ is red and second flash at t_2_ is blue, then the particle color at the location corresponding to t_1_ will be blue and vice versa for the two-color case. This is because the particle at t_1_ blocks the red light and moves before the blue light fills in the shadow. The power of shadow PIV imaging (PSV) was demonstrated by Estevadeordal and Goss in previous research^23^. For two-color cases we use the blue and red flashes to minimize cross-talk between them, as these wavelengths are the most separated of the three colors. However, getting sufficient energy in the blue LED can be a challenge^27^. Cross-contamination will of course depend both on the spectral content of the illuminating light and the shape of the band-pass filter in the Bayer matrix on the sensor. This greatly facilitated the separation of color channels without any pre-processing of the images, as is clearly shown in Fig. 4(b–d).

**Figure 5:**
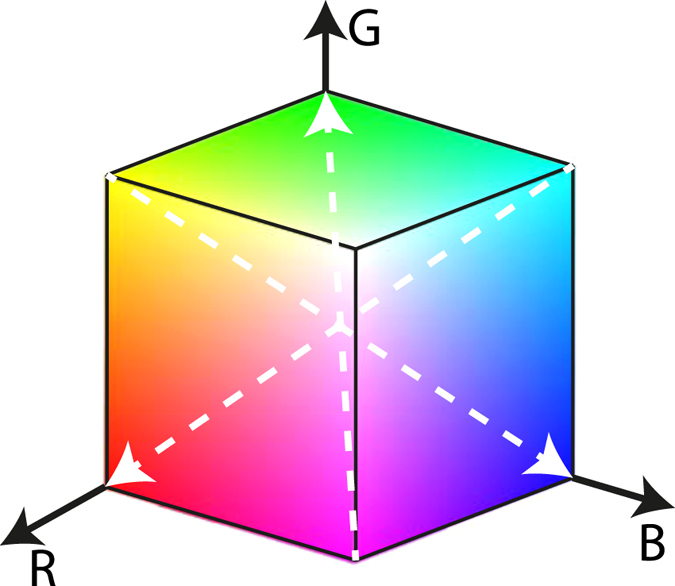
The RGB color space represented in Cartesian coordinates. The white dashed arrows link the corresponding negative colors that are captured in the images.

**Figure 6:**
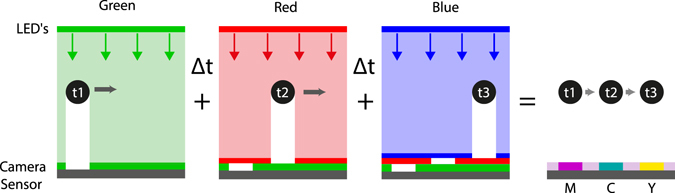
Schematic representation of the color time-coding approach used for the CMOS sensors. As the particle travels, the colored lights are flashed at different times, leaving their corresponding shadowed pixels recorded in the camera sensor. The final result is an image of the negative colors for each time step, i.e. green flash marks the particle location by magenta pixels, red by cyan and blue by yellow.

For a second implementation of the technique, of primary focus herein, we exploited all three color-channels on the sensors of the cameras, by using a three color LED illumination. Each color of the LED light was pulsed separately, e.g. in a green-red-blue sequence, thereby encoding three different time-instants, t_1_, t_2_ and t_3_ on the same image. All three flashes occur within the same exposure time of the cameras, capturing three different spatial positions of each particle. The particle-color denoting each time step on the sensor is detected as the corresponding negative color in the RGB color space, as depicted in Fig. 5. From this diagram, we observe the yellow pixels in Fig. 6 correspond to the blue flash. Similarly, the cyan and magenta pixels in Fig. 6 correspond to the red and green flashes, respectively. In both implementations (two and three colors), the experiments were performed by varying the intervals between the color flashes (Δt) from 1300 µs for the slowest vortex ring to 500 µs for the fastest one.

### Color crosstalk and Chromatic aberrations

Using multi-color illumination introduces a bias error to the particle locations, which arises from the slightly different refraction of the three wavelengths of light, when they pass through the thick plexiglass wall of the tank. Furthermore, color crosstalk between the RGB channels due to mismatch of the emission bandwidth of the LED lights and the transmission spectra of the Bayer filter array used in the camera sensor, can negatively affect the results. These effects were studied in great detail by McPhail *et al*.^24^ for 2D PSV with a 3-colored LED illumination system, where they showed how to implement chromatic corrections to reduce the bias error in the system.

**Figure 7:**
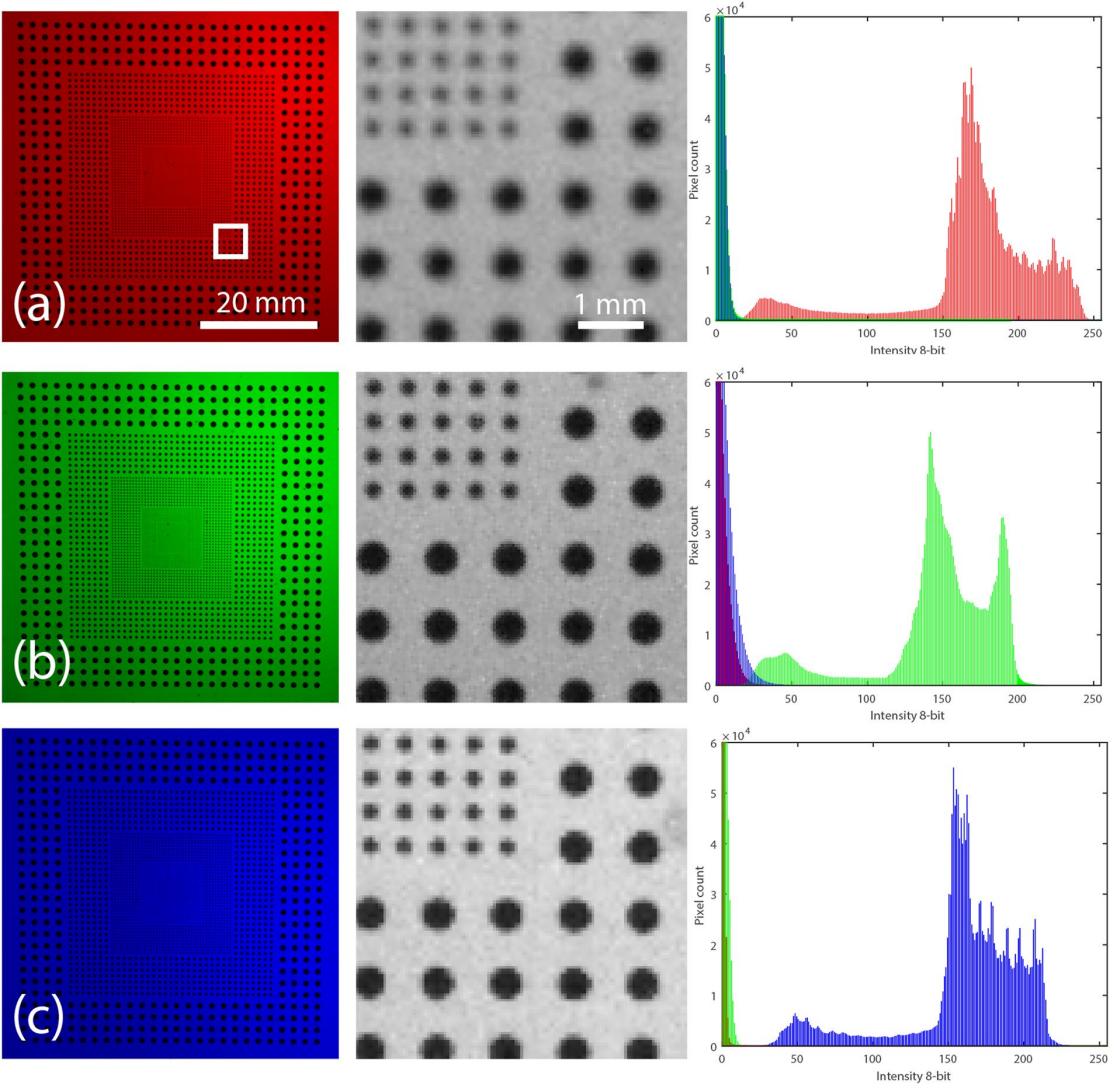
Images of the dotted calibration glass slide inside the water-tank, back-illuminated by red (**a**), green (**b**) or blue (**c**) LED flashes. The middle column images are magnified sections of the dots corresponding to the white square in panel (**a**). On the right column we show the corresponding RGB intensity histograms.

In order to quantify the above sources of systematic error, we use a glass calibration plate which contains regular arrays of differently sized black dots. This target is placed inside the water-tank at the location of the vortex ring. Separate images are then captured by flashing the background diffusers with a single color at a time, allowing us to quantify the shift in the position of the dots due to chromatic aberration and to quantify the crosstalk between the color channels, as is depicted in Fig. 7.

From the histograms in Fig. 7 we observe that color crosstalk is quite limited in our setup, which allows us to remove any cross talk by thresholding at a low intensity levels, without losing any details of the particles.

**Figure 8:**
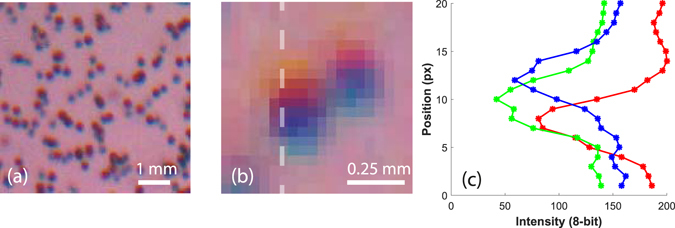
(**a**) Particle images with the three color LED’s flashed simultaneously, i.e. with zero time-delay. (**b**) Close-up of two particles, which shows clearly the effects of the chromatic aberrations in one of the most affected areas, near the edge of the image. (**c**) The color profiles of a single particle, taken along the dashed line in (**b**). One can clearly see the vertical shifting of the colored shadows, due to the chromatic aberrations.

McPhail *et al*.^24^ report adjusting for the chromatic aberrations of the colors by a “Zero-time-delay” correction, which consists of triggering all of the color flashes simultaneously. We expand their method to our 3D technique, capturing the “Zero-time-delay images” of a typical particle field, for all the cameras at the same time, so the particles do not move between the different colored flashes. This allows us to reconstruct the three particle fields and calculate the two 3D displacement-vector fields, between the green and red flash, as well as between the red and blue. Without chromatic aberrations, no displacement of particles should be observed. However, it is clear from the images in Fig. 8, that some areas of the images are highly affected by the chromatic aberrations and the particle locations shift between the flashes. At the edge of the image we have a displacement of 2 to 3 pixels between the colors, which corresponds to around 80 to 120 μm. The images are then loaded and processed by the tomographic PIV algorithm with the same parameters as for the moving particles. This chromatic-aberration shift is primarily related to the angle of viewing through the wall and is therefore a slowly varying function over the image area and should remain fixed throughout the experiments, if the cameras are not moved. Example of these displacement fields are shown in Supplementary Fig. S1. The magnitude of this effect is also different between green-red vs red-blue particle fields. The final velocity fields are corrected by subtracting these error velocity fields from their corresponding final velocity fields.

## Results

### Pre-processing of the images

**Figure 9:**
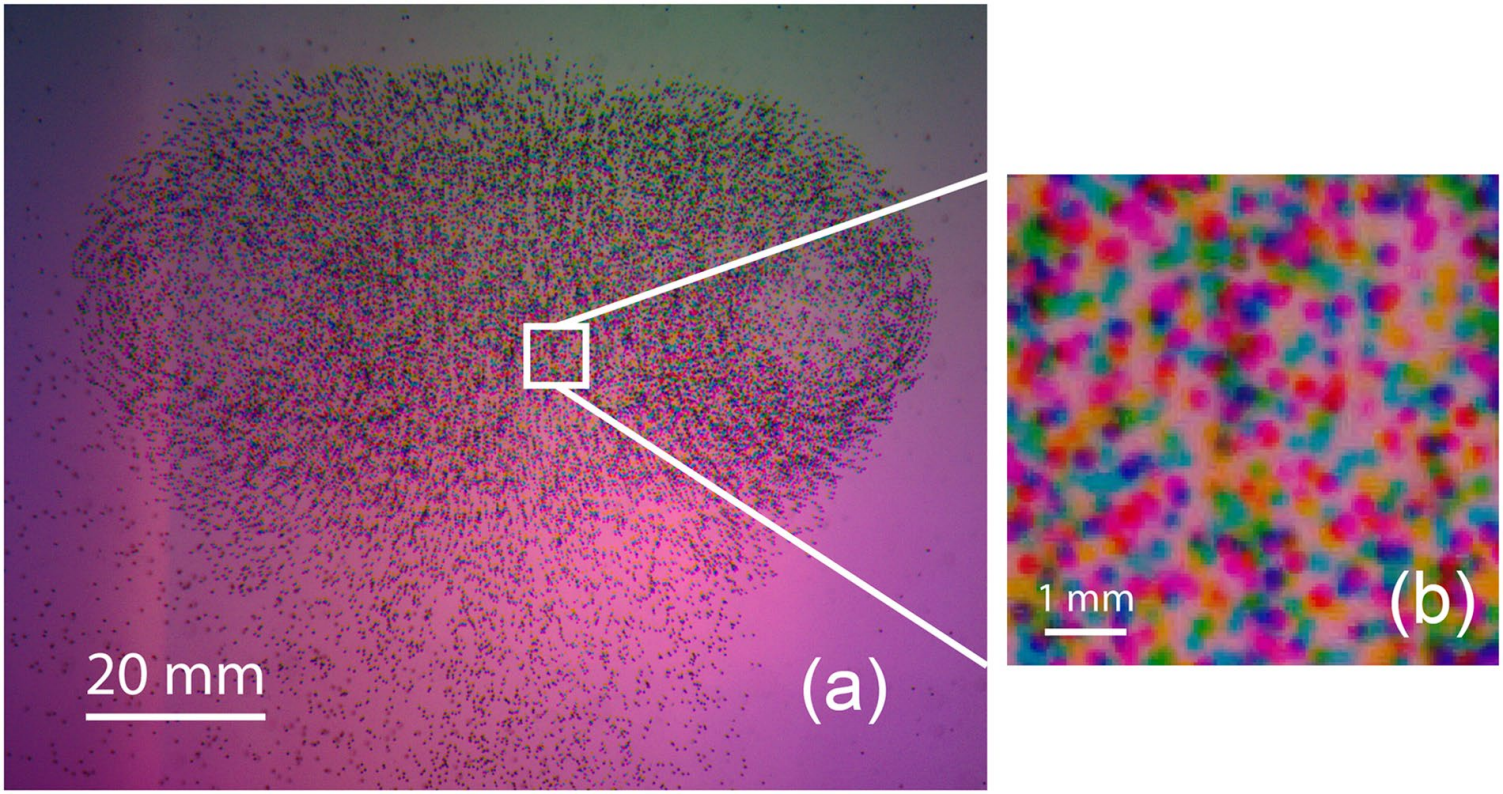
(**a**) Captured image from the bottom central camera (phone 3). In this image we clearly observe the three different colored shadows and the vortex ring structure in a “mushroom” shape. (**b**) 9x magnification of a sampled area, inside the white square.

Figure 9(a) shows a typical smartphone image captured by our method, revealing the vortex ring structure demarcated by the seeding particles in a “mushroom” shape. The tracer particles average image density within the vortex is N = 0.015 ppp (particles per pixel) considering the three RGB color channels, this translates into N = 0.005 ppp for each time step. When estimating the particles per pixel density it is important to keep in mind the relatively large size of the particles used in our setup. Using a color sensor one needs more pixels to effectively pin down the particle center, than is required for a regular monochrome PIV sensor setup. Additionally one can obtain the source density (N_s_), i.e. the fraction of the image occupied by particles^28^, this translates to N_s_ = 0.3. Reducing the particle size makes it possible to increase the particle per pixel density (N), while keeping the source density (N_s_) constant. For this technique we estimate that the lower limit of each particle size is 4 pixels to avoid false color due to pixel binning, which can arise from the interpolation of the Bayer filter array, as previously mentioned. The single channel concentration in ppp is relatively low compared to traditional tomographic PIV, but is necessary to separate the particles for each color channel without devising too much overlap that may affect the color separation and low quality reconstruction.

The maximum particle displacement between the flash instants was approximately 15 pixels, as measured in the fastest regions around the vortex core. As observed in Fig. 9(a,b), the background exhibits significant large-scale spatial gradients in the color intensities. This inhomogeneous background in the picture occurs due to the different locations of each of the LEDs behind the projection lens and a slight unbalance in the intensity of each colored light. Owing to the fixed camera lenses, with constant focal length and aperture, the depth of focus is also fixed. These inflexible optics in addition to the size of our tank, limits the accessible region of interest (ROI). Thus, the 38 Mpx image area has to be cropped to a ROI of 2500 × 2000, i.e. an active area of approximately 5 Mpx. Considering that most of the commercial cameras used for PIV have a typical resolution of 4 Mpx or less, for configurations where the full image resolution of the smartphones can be used, the 40 Mpx would be almost ten times larger than the specialized equipment.

**Figure 10:**
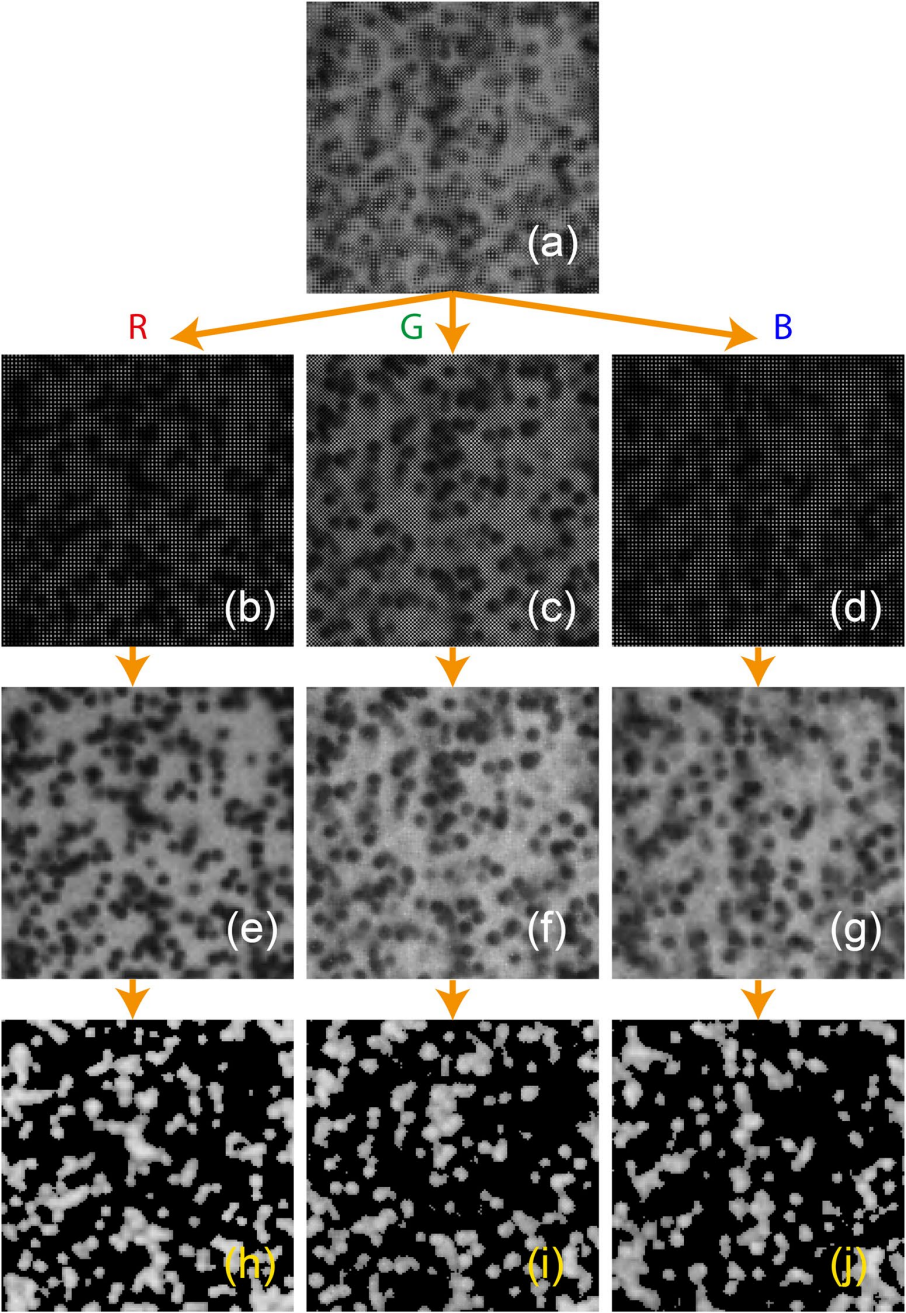
Steps for preprocessing of the raw image file, showing the same region as in Fig. 9(b). (**a**) The raw image from the Bayer filter array in gray-scale intensity. (**b**–**d**) Separation of the color channels based on pixel location in the array, with other pixels filled by zeros. (**e**–**f**) Resulting images after demosaic interpolation (Malvar *et al*.^29^). (**h**–**j**) Final images for each color after background subtraction and filtering by local standard deviation levels.

We use the DNG image files of the particles, which are acquired using the GRBG Bayer filter array present on the smartphone sensor, see Fig. 10(a), before the interpolation is performed to generate the three color channels. We first show, in Fig. 10(b–d), the three separate images for each color array, assigning a 0 level of intensity for the other color pixels. Each of these images is then interpolated using the demosaic method proposed by Malvar *et al*.^29^, which consists of a gradient-corrected bilinear interpolation, creating sharper edges than a simple bilinear interpolation, see Fig. 10(e–g).

**Figure 11:**
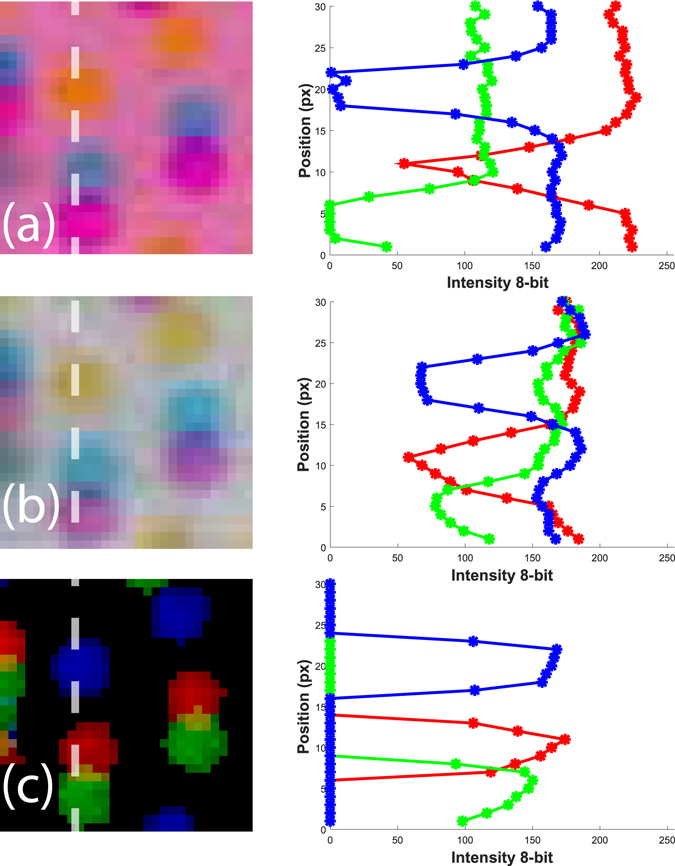
(**a**) Cropped portion of two particles from the raw captured image with the default color interpolation by the smartphone, (**b**) the interpolated raw image after separation of each channel array and combining them for clarity. (**c**) Inverted image after background subtraction and filtering by local standard deviation in each subregion, combining the color channels for clarity. For comparison, the color intensities are plotted on the right side, along cuts through the image, marked by the dashed line.

The effects of the inhomogeneous background-color intensities can be minimized with additional image pre-processing steps in the following manner. First, we record several images of the same 3-color pulse-lighted background without any particles in the field of view. We use these images to subsequently normalize the background and intensities of the particle shadows. We do this by treating the background RAW image-files in the same way of separation and demosaic interpolation as the particle images, thus, obtaining the background reference frames for each color. Taking the local average background for each color and inverting the color intensities of both, the averaged background and particle images, allow us to subtract their intensities, resulting in a more uniform background and enhancing the particle signals. To further reduce background noise and any far-out-of-focus particles, the image is divided in subregions of 100 × 100 px to obtain their mean intensity (Ī) and standard deviation (σ) locally. Pixels with intensity levels lower than Ī + 3σ are filtered out inside each region. The threshold is strict, but real particles have a much higher intensity than the background at this point, Fig. 10(h–j). An animation of the final split of the three frames played in sequence can be found in the online Supplementary Video S1. The pixel intensity curves for the processing steps of the images are explained in Fig. 11.

### Tomographic PIV reconstruction and correlation procedures

Once the images for all four cameras are pre-processed and split up into the separate color channels, they are imported, together with the calibration images, into the commercially available software Davis 8.2.2 from LaVision. To the best of our knowledge, there is currently no open-source alternative for a tomographic PIV software, since Davis software is already heavily optimized and is in a mature state, offering sophisticated reconstruction algorithms utilizing GPU’s parallel computing. Other algorithms for 3D reconstructions and cross-correlations have been published, but adapting them for efficient computing is a challenge in its own right. Any current Tomo-PIV system will therefore bear the cost of this software, irrespective of the hardware.

**Figure 12:**
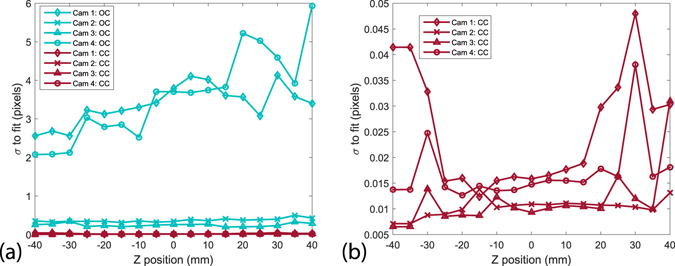
(**a**) Standard deviation of the original calibration fit (OC, cyan color), and corrected calibration (CC, red color) after three iterations of the self-calibration algorithm as a function of depth position z. (**b**) Detailed plot of the corrected calibration.

The initial calibration is carried out on all 17 shifted images of the calibration plate, the error is shown in Fig. 12(a). We use a third order polynomial for the fit model. The raw calibration results in a large errors for the two off-axis cameras (1 & 4 in Fig. 1b) with a standard deviation of the order of 3.5 pixels, while the two center cameras have subpixel deviations. We note that cameras 1 and 4, view the calibration target from a much larger angle than the cameras at the center. However, this is greatly improved by subsequently performing a self-calibration, where the reconstructed particles are triangulated and used directly to correct the calibrations via disparity maps^30^. This reduces the calibration errors for all cameras below 0.05 pixels after three iterations, as shown in Fig. 12(b).

Hereafter the images are uploaded to the DaVis program and assigned for each camera and time-step. Then particle locations are reconstructed in the 3D volume, plane by plane, with the Fast MART algorithm, which includes a MLOS initialization and 10 iterations of CSMART^7^. As a result, the volume of 200 × 160 × 120 mm^3^ is discretized with 3044 × 2436 × 1822 voxels, resulting in approximately 3500 voxels/mm^3^.

**Figure 13:**
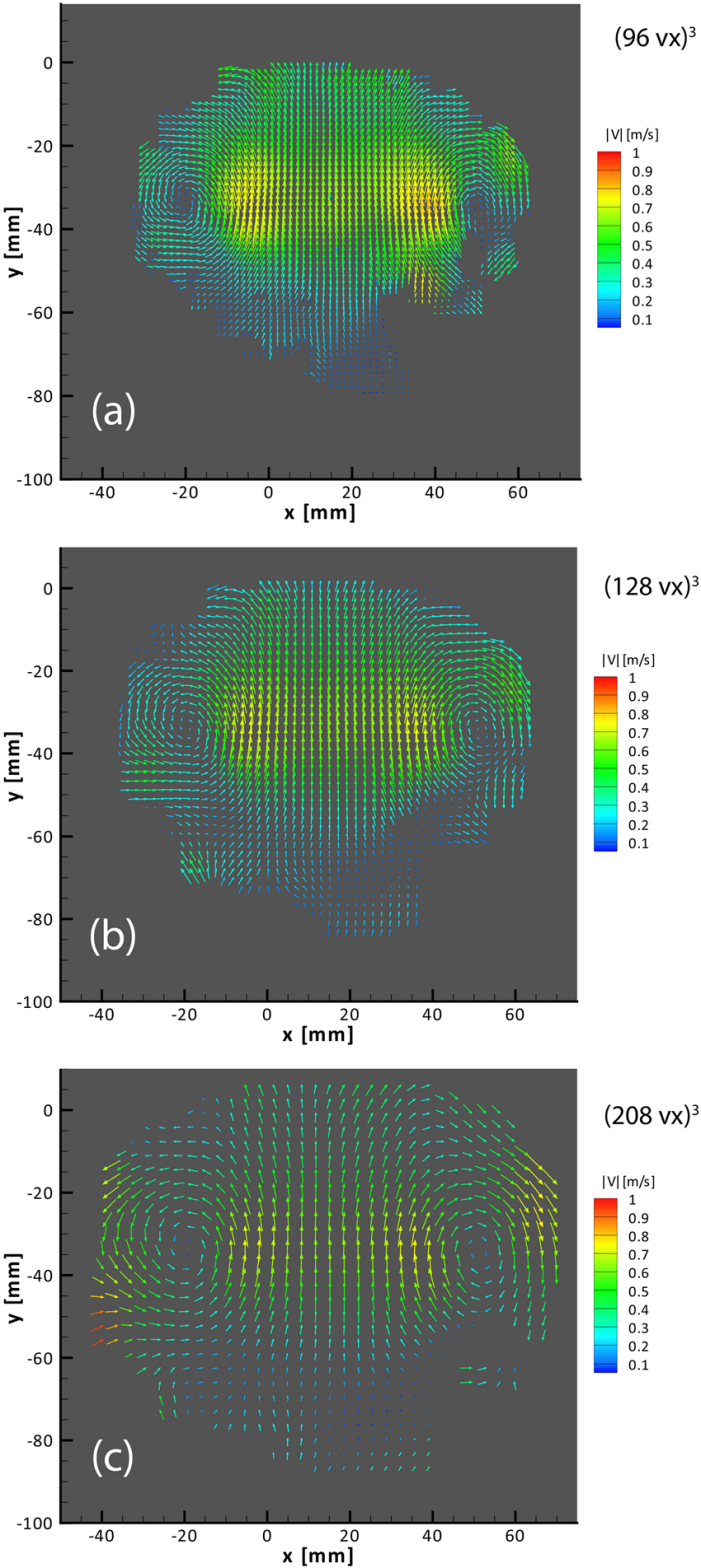
Instantaneous velocity field vectors obtained after direct cross-correlation at the plane z = 0 mm captured with Δt = 1000 μs, comparison between 96 (**a**), 128 (**b**), and 208 (**c**) voxel^3^ interrogation volumes.

We proceed to do Direct Cross-Correlation between the different time steps (t_1_-t_2_ and t_2_-t_3_). This is done for three different sequences of reduced interrogation volume sizes. Figure 13 compares the velocity results of the three different sequences, i.e. 512 → 320 → 208 voxel^3^, 512 → 256 →  → 128 voxel^3^ and 352 → 128 → 96 voxel^3^. Each size reduction step in the correlation is performed with a 75% interrogation volume overlap and iterated with 8 passes at the final resolution. Gaussian smoothing is also used between iterations to improve the quality of the vector field. The purpose of initializing the process with bigger interrogation volumes is to obtain a rough initial estimation of the velocity field, while refining it using the general direction of the flow when the interrogation volume is reduced. This will allow us to obtain a more detailed and accurate velocity field resulting on a 3D grid of 3.3, 2.1 and 1.55 mm vector pitch respectively. At this point the 3D Zero-time-delay correction for chromatic aberration is subtracted from the resulting velocity field. It is obtained using the same parameters for reconstruction and direct cross-correlation used for the moving particles, as explained in an earlier section. The Zero-time-delay velocity field is presented in Supplementary Fig. S1. A comparison of the results before and after the color aberration correction is available in Supplementary Fig. S2.

Comparing the 2-D cuts through the velocity fields for the 3 different-volumes sizes, we conclude that the case of 128 voxel^3^ is most suitable for this study, as it shows the detailed velocity field without the loss in accuracy observed for the smallest interrogation volume of 96 voxel^3^, nor losing some details at the bigger volume of 206 voxel^3^. Bad vectors outside the well-seeded region have been removed by masking.

**Figure 14:**
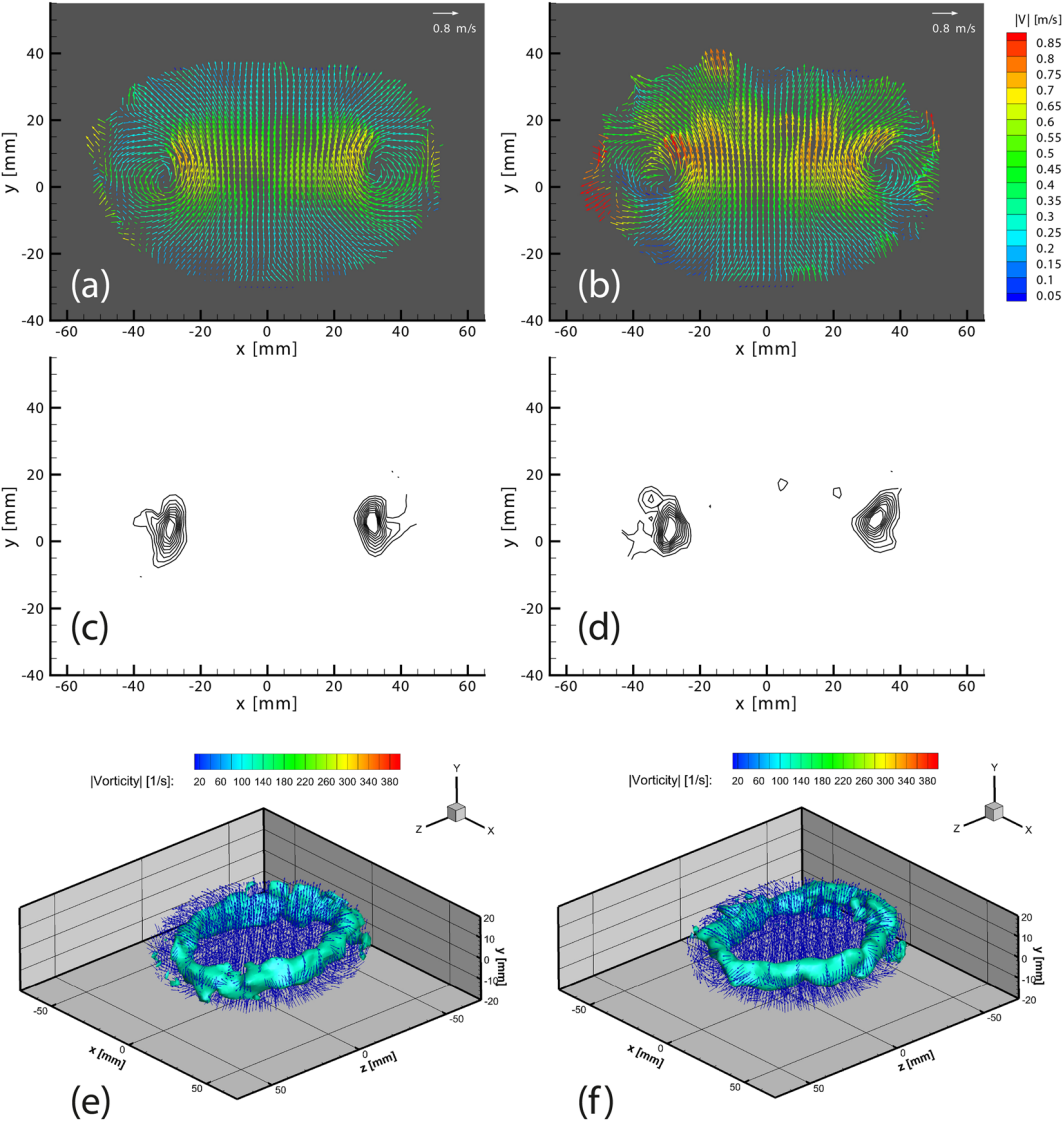
Instantaneous velocity fields for the density-matched case with Re = 24,000 for consecutive time steps in the plane z = 0 mm for (**a**) t_1_-t_2_ correlation (G-R) and (**b**) t_2_-t_3_ correlation (R-B), both with Δt = 1300 μs. The corresponding isocontours of vorticity magnitude ranging from 70 to 230 s^−1^ for (**c**) G-R correlation and (**d**) R-B correlation. The corresponding vortex ring visualized in 3D by the isosurface of vorticity magnitude 110 s^−1^ as well as every fifth vector of the instantaneous velocity field (**e**) G-R correlation (**f**) R-B correlation.

Additionally, we compare the instantaneous velocity fields for different air-impulse pressures, i.e. different circulation strength and translational velocities of the vortex rings. This required reducing Δt from 1000 to 500 µs for the faster ring in the case of pure water, and using Δt = 1300 µs for the aqueous sodium chloride solution. In all cases the method reproduces accurate velocity fields. Results for Δt = 1000 and 500 µs are presented in Supplementary Fig. S3. For the case of Δt = 1300 µs an increase in velocity magnitudes in Fig. 14(a) vs (b) can be observed for the successive velocity fields due to the accelerating motion. The core structure can be extracted from the vorticity field, such as the isocontours of vorticity magnitude, ranging from 70 to 230 s^−1^ plotted in Fig. 14(c,d), which allows us to locate the core of the vortex. Furthermore, Fig. 14(e,f), shows 3-D perspective plots of the isosurfaces of the 110 s^−1^ vorticity magnitude and the surrounding 3D-3C velocity vectors, in order to highlight the ring structure in 3D.

The Reynolds number of the flow can be approximated as *Re* = *Γ/ν* (see Gharib *et al*.^31^), where the maximum circulation (Γ) value for the pure water cases results in Re = 44,000 and 56,000 for Δt = 1000 and 500 µs, respectively. The aqueous sodium chloride solution results in a Re = 24,000 using Δt = 1300 µs shown herein, since the salt increases the kinematic viscosity. Our results show a similar vortex ring structure as reported in previous experimental studies^3, 31, 32^.

### Estimating the acceleration field

Our three-color measurements give three particle locations, which allows us to measure two subsequent velocity fields, but also opens the possibility of calculating the acceleration of the fluid elements. An estimation of the instantaneous local acceleration field ∂**u**/∂t can be approximated with a first order finite difference of the two instantaneous velocity fields. This has been demonstrated by McPhail *et al*.^33^ for their planar PSV experiments. However, having the full 3-D velocity we can also obtain the advected acceleration term **u**•∇**u**. Supplementary Fig. S4 shows the resulting local and advected acceleration fields in a cut through the vortex ring. However, keep in mind that the errors in the velocity field are enhanced by the derivatives and for a detailed study of the accelerations one should optimize the velocity measurements and increase the spatial and temporal resolutions.

We also note that having three subsequent images of the particles opens up new possibilities for the correlation method, where third-order correlations can be used to refine the velocity estimates^34^. This has been highlighted as a promising avenue for improvements, in the recent review of Westerweel *et al*.^2^.

### Circulation and continuity verification

**Figure 15:**
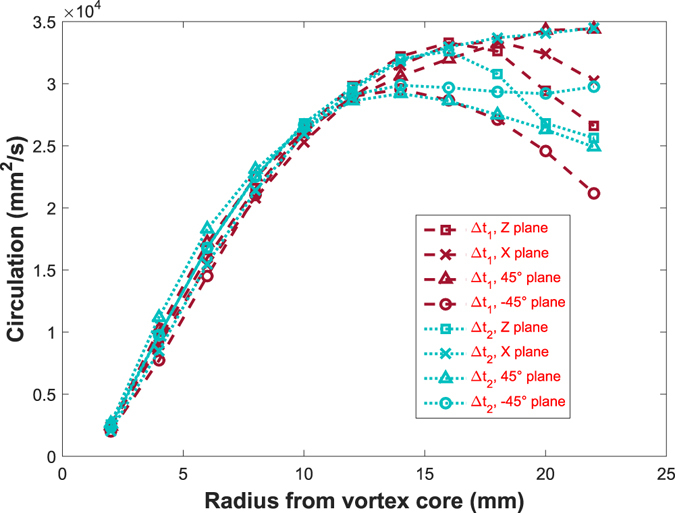
Circulation Γ as a function of radius from vortex core, in four different planes, for t_1_-t_2_ correlation (red) and for t_2_-t_3_ correlation (cyan), with Re = 24,000.

Furthermore, to test the consistency of the data, we can obtain the circulation Γ at different radii from the core of the vortex ring. This is done in different planes from the volumetric data, by integrating the vorticity contained within circular discs of radii varying from 2 to 22 mm. We compare planar cuts through the vortex ring in the planes perpendicular to the x- and z- axes, as well as the planes at ±45° between them, obtaining cross-sections that pass through the axis of symmetry. From Fig. 15, we observe that the circulation for Re = 24,000, has the same structure for all the cuts taken at the different azimuthal angles for both successive velocity fields. Circulation is close to constant between the two fields. This is particularly true for radii under 12 mm, which is close to the size of the vortex ring. For larger radii limitations from the masking on the outer edge make the data less accurate in some of the planes. Keep in mind that the y-z plane, is only viewed edge-on by the front cameras (2 & 3 in Fig. 1a) while viewed from 45° angle in the other two cameras (1 & 4). This conservation of the circulation supports the consistency of the results. The corresponding results for the Re = 44,000 and 56,000 are presented in Supplementary Fig. S5.

**Figure 16:**
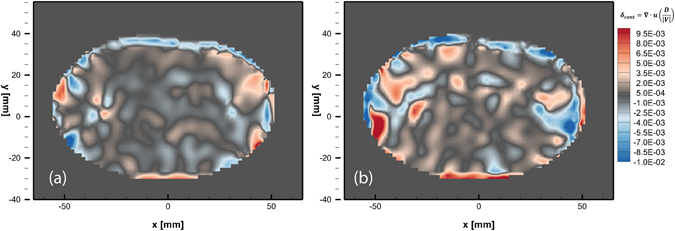
Contours of normalized residuals from the continuity equation, ∇·**u**, for (**a**) first time-step (G-R) and (**b**) second time-step (R-B), taken at the plane Z = 0 mm.

Additionally, we check the consistency of the results by verifying conservation of mass through the closure of the continuity equation for incompressible flows (∇·**u** = 0), where the residual should be near to 0. This value of continuity residual δ_cont_ is then normalized by the inverse of a characteristic time scale (τ = 0.081 s) defined as the ratio of the vortex ring diameter (D = 0.065 m in this case) divided by vortex ring maximum magnitude of velocity, |V| which is here ~0.8 m/s. Figure 16 shows the resulting normalized residual in a single plane for the two consecutive velocity fields. The mean normalized residual of the whole volume considering only their absolute values is $\overline{{\delta }_{cont}\,}=1.43\times {10}^{-3}$ with a standard deviation $\sigma =\,2.20\times {10}^{-3}.\,$

### Comparison with concurrent Stereoscopic PIV measurements

In addition to the above consistency checks of continuity and constancy of the circulation, we have also carried out a set of experiments which compare the tomo-PIV results with simultaneous Stereoscopic PIV measurements. This LaVision system consists of two 4-Mpx Imager-Pro-X dual-frame CCD cameras to capture two time steps with Δt = 1300 μs.

These cameras are placed just above cameras 1 and 4, (see sketch in Fig. 1a) looking at the same ROI of plane Z = 0 mm with 50 mm Nikkor lenses in a Scheimpflug configuration to focus both cameras on the same plane. We use the largest possible aperture to reduce the depth-of-focus. The same LED illumination system must be used to acquire the tomo and stereo PIV data concurrently. The backlit volumetric illumination will add defocused particles to the images captured in the stereo PIV system. For this reason it is necessary to preprocess the stereo images by inverting their intensities and dividing the 4 Mpx region into subregions of 100 × 100 px to obtain their mean intensity (Ī) and standard deviation (σ). Pixels with intensity levels lower than Ī−σ are filtered out minimizing defocused particles and creating a uniform background. The particle density in the stereo-PIV images is therefore much lower than for ideal conditions, as is shown in Supplementary Fig. S6.

**Figure 17:**
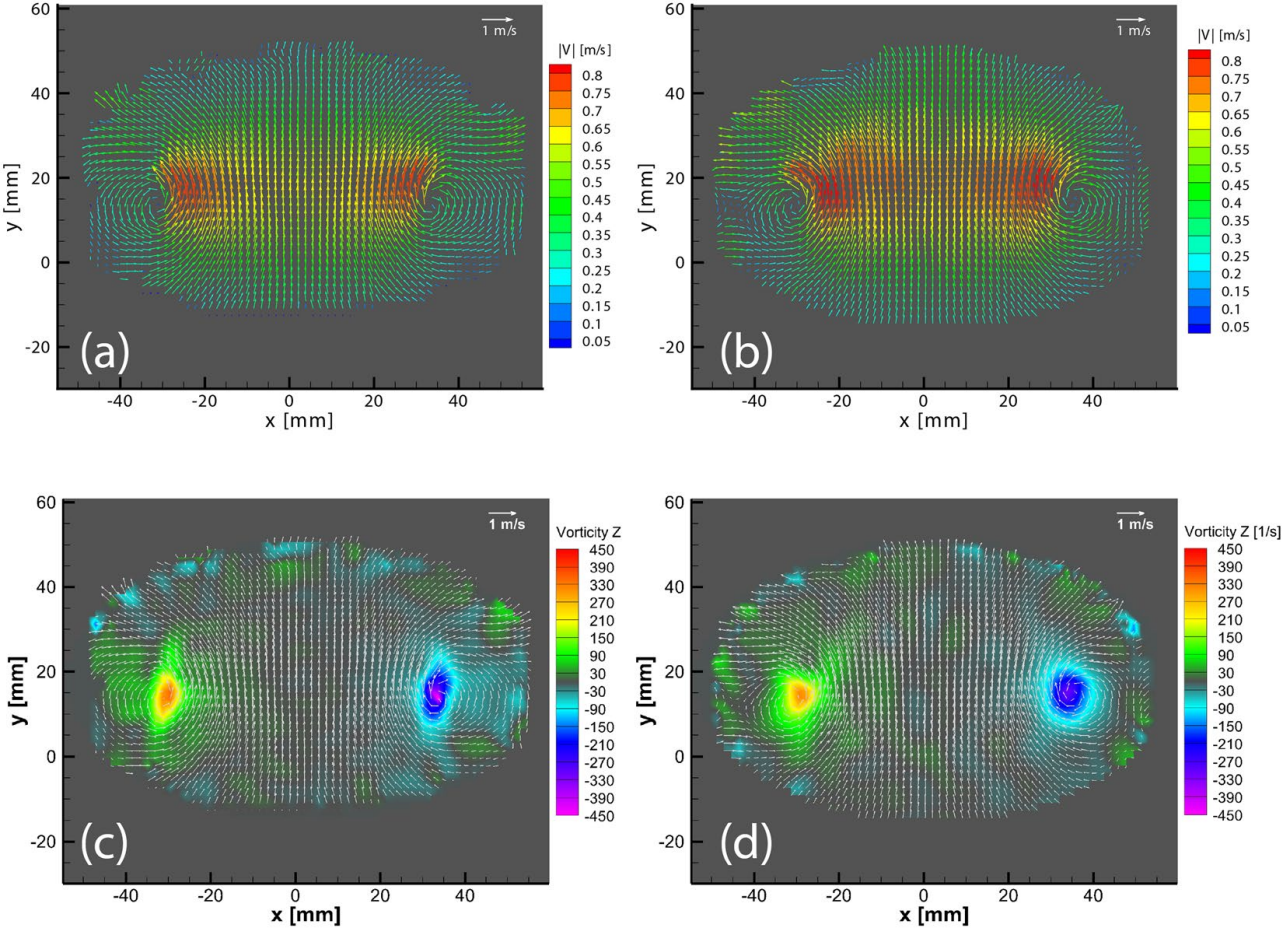
Side by side comparison of an instantaneous velocity field (Δt = 1300 μs) between (**a**) stereo PIV system vs (**b**) smartphone Tomo PIV system, colored by the velocity magnitude. Comparison of contour plots of vorticity in Z between (**c**) stereo PIV system vs (**d**) smartphone Tomo PIV system (velocity vectors are included for further orientation reference). All images are presented in the plane Z = 0 mm.

The stereo PIV system dual frame is synchronized with the LED’s using a digital delay generator, thus allowing to capture the same two time steps for the Tomo and Stereo systems. The results obtained from the stereo PIV system provides a benchmark of the three velocity components in a single plane (Z = 0 mm). A side by side comparison for the velocity fields and vorticity in the Z = 0 plane is presented in Fig. 17. We can clearly observe that the main features of the vortex ring, such as the structure and magnitude of the velocity field and vorticity, are reproduced with a good degree of accuracy in our proposed system.

Keep in mind that for this setup one can expect the Tomo system to give more accurate results, as the seeding density is lower than the one used for typical stereo PIV, where a thick laser-sheet illuminates the plane of interest.

**Figure 18:**
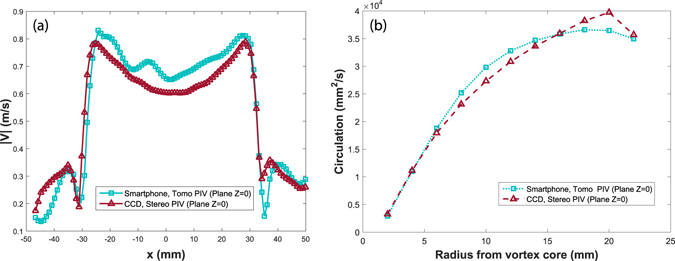
(**a**) Comparison of the velocity magnitude along a cut through the vortex cores in the plane Z = 0 mm for our Tomo-PIV system (cyan) vs 2-camera stereoscopic PIV system (red). (**b**) Comparison of the circulation Γ as a function of the radial distance from the center of the vortex cores.

For further comparison, we plot the 3D velocity magnitude along a horizontal cut through the center of the vortex cores, as is shown in Fig. 18(a). The figure shows close similarity of the velocity profiles between the two independent measurements. The largest difference in velocity magnitudes is on the edges, primarily due to slight shifting in the location of the vortex cores, where the velocity gradients are largest. By normalizing these differences with respect to the maximum magnitude of ~0.8 m/s, this yields a relative error of less than 15%. Supplementary Fig. S7 compares these deviations over the entire overlapping area of the plane at Z = 0. It shows that 84% of the total area has an error of 20% or less. We emphasize that the largest errors are due to slight offset in the location of the centers of the vortices. Similarly we assess the consistency of the results, by calculating the circulation obtained from both experimental techniques showing very similar results in Fig. 18(b), with deviations of less than 8%.

### Implications for hardware cost

In a conventional Tomographic PIV system the hardware represents the largest expense, with several options on camera frame-rates, sensitivity and illumination systems available. A system comprising of four dual-frame CCD cameras (not high speed) with high sensitivity, 200 mJ double pulsed laser capable of illuminating a similar volume of interest as the one studied in this work, a time unit control and other optical components can add up to $200,000 USD.

In contrast, the overall cost of the hardware used in this investigation is roughly $6,000 USD, which represents a dramatic decrease of 33X in the hardware investment. Such cost includes four smartphones, three LED’s for each color channel (nine in total), three LED drivers (one for each color) and a digital delay generator. Finally, we point out that a further reduction in the total cost could be achieved, by replacing the LED drivers and digital delay generator, which corresponds to approximately 60% of the total cost, with low-cost microcontrollers and in-house designed drivers.

## Conclusions

Tomographic PIV is becoming the preferred technique for 3D quantification of turbulent flow fields. However, the hardware required for tomographic PIV is prohibitively expensive, primarily due to the need for multiple high-resolution CMOS or CCD cameras as well as a powerful pulsed light source, which can illuminate a volume of the flow regime. This is usually accomplished with lasers, which are expensive and additionally pose safety issues in the lab. The operations, synchronization and control system are also not trivial. Finally, the software required to reconstruct the 3-D particle distribution and calculate the velocities through cross-correlations demands specialized algorithms which are still being optimized to overcome the tremendous computational cost.

In the current paper we have demonstrated the feasibility of using low-cost smartphone cameras and high-power LEDs to replace the currently used specialized high-cost Tomo-PIV hardware. We successfully reconstructed the flow field of a vortex ring at Re ∼ 24,000, 44,000 and 56,000. We overcome the slow frame-rate of the smartphone-cameras by encoding two or three time-steps on the same frame, by forming colored shadows of the particles, using red, green and blue LED light pulses. This system can capture fast moving flow, as the time-step between pulses only needs to be 5–10 times the pulse duration. In our setup this pulse duration is 80 µs and could be reduced by adding more LEDs for each color. The full capturing speed potential of this system can be tested in higher Reynolds number systems.

The main limitation encountered herein is the lack of interchangeable lenses and fixed aperture in the smartphones optics. However, zoom lenses are already beginning to appear on smartphone cameras and will soon allow their application in more general configurations. The synchronization of the cameras is here accomplished with a Wi-Fi router, which has shown to introduce random delays, which may interfere with precise timing of lights and camera exposures. This we have overcome by opening the shutters for 1 second and using synchronized flashes. However, using previously synchronized internal clocks, or even the GPS signal clock might overcome this problem. This will become even more critical if one uses the video-capabilities of these cameras, which are improving rapidly, both in pixel resolution, as well as higher frame-rates for “slow-mo” recording^25^. Using 4k-video and frame-straddling offers tantalizing new opportunities.

The underlying rational for using smartphone cameras in this work, is to exploit the economics of scale by piggy-backing on the hundreds of millions of consumers using smart-phones. The nature of electronics manufacturing often reduces the cost of consumer products by a hundred-fold, compared to specialized scientific instruments. Herein, we have used smartphone cameras to acquire the images and subsequently transferred them to dedicated computers running specialized LaVision software to extract the 3-D velocities. One can envision taking advantage of the growing computing power in the smartphones themselves for the data reduction, to realize a portable tomographic-PIV system becoming feasible in the near future, for industrial, scientific and educational applications.

## Electronic supplementary material

Animation of particle motion (single camera)

Supplementary Figures
