# Supplementary material for: Tomographic Particle Image Velocimetry using Smartphones and Colored Shadows
Source: Sci Rep. 2017 Jun 16;7:3714. doi: 10.1038/s41598-017-03722-9 (PMC5473851; doi:10.1038/s41598-017-03722-9)
Supplement: Supplementary file 2 — Supplementary Figures [file 41598_2017_3722_MOESM2_ESM.pdf]

Tomographic Particle Image Velocimetry using Smartphones and Colored Shadows

Andres A. Aguirre-Pablo[1], Meshal K. Alarfaj[1], Er Qiang Li[1], J. F. Hernández-Sánchez[1], Sigurđur T. Thoroddsen[1]

[1]Division of Physical Sciences and Engineering, King Abdullah University of Science and Technology (KAUST), Thuwal 23955-6900, Saudi Arabia

SUPPLEMENTARY INFORMATION

Supplementary Video S1.

Animation of the three continuous time steps after pre-processing the particle images. The color-channels from one of the smart-phones are played back and forth. The particle motion around the vortex ring can be clearly observed.

Tomographic PIV reconstruction and correlation procedures

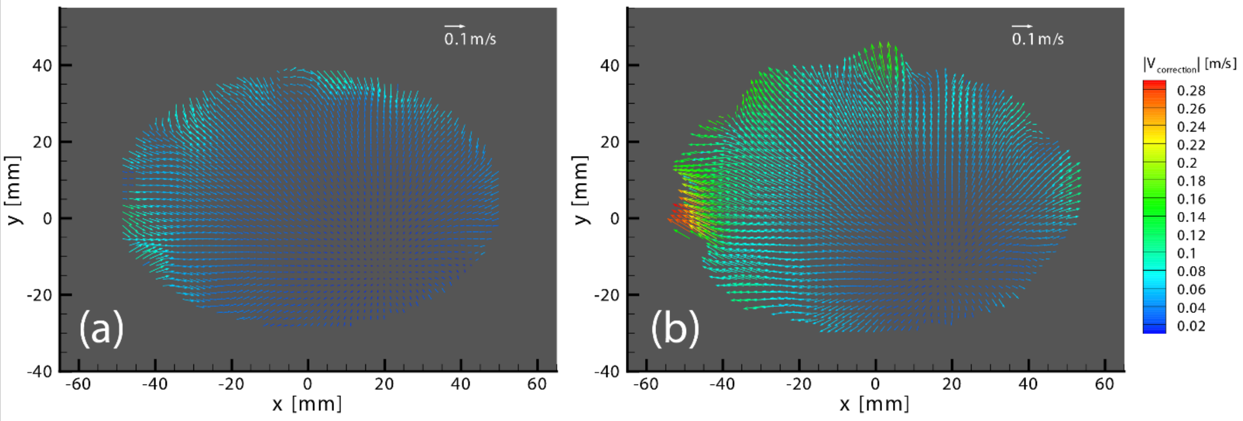

Supplementary Figure S1. Velocity bias errors due to chromatic aberration. They are obtained from the Zero-time-delay images (all colors flashed at the same time) for (a) t1 – t2 correlation (Green-red) and (b) t2 – t3 correlation (Red-Blue). The arrows in both cases are colored by their vector magnitudes. Both results are presented in plane z=0 mm. Note that most of the fields have aberration magnitudes less than 0.1 m/s.

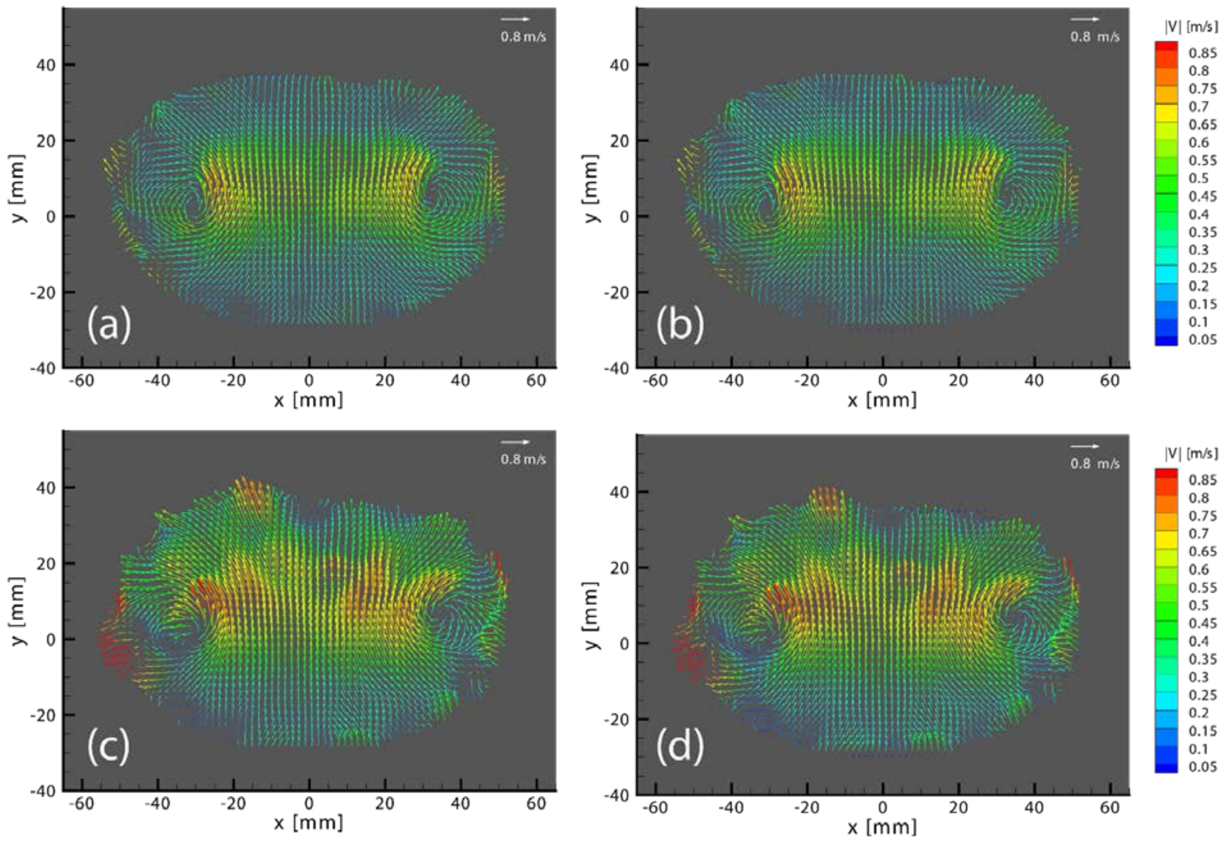

Supplementary Figure S2. Side by side comparison of the original velocity field (a,c) vs the chromatic aberration corrected velocity field (b,d). Top figures (a,b) correspond to the t1 – t2 correlation, bottom figures (c,d) correspond to the successive t2 – t3 correlation. All figures belong to the Re=24,000 case.

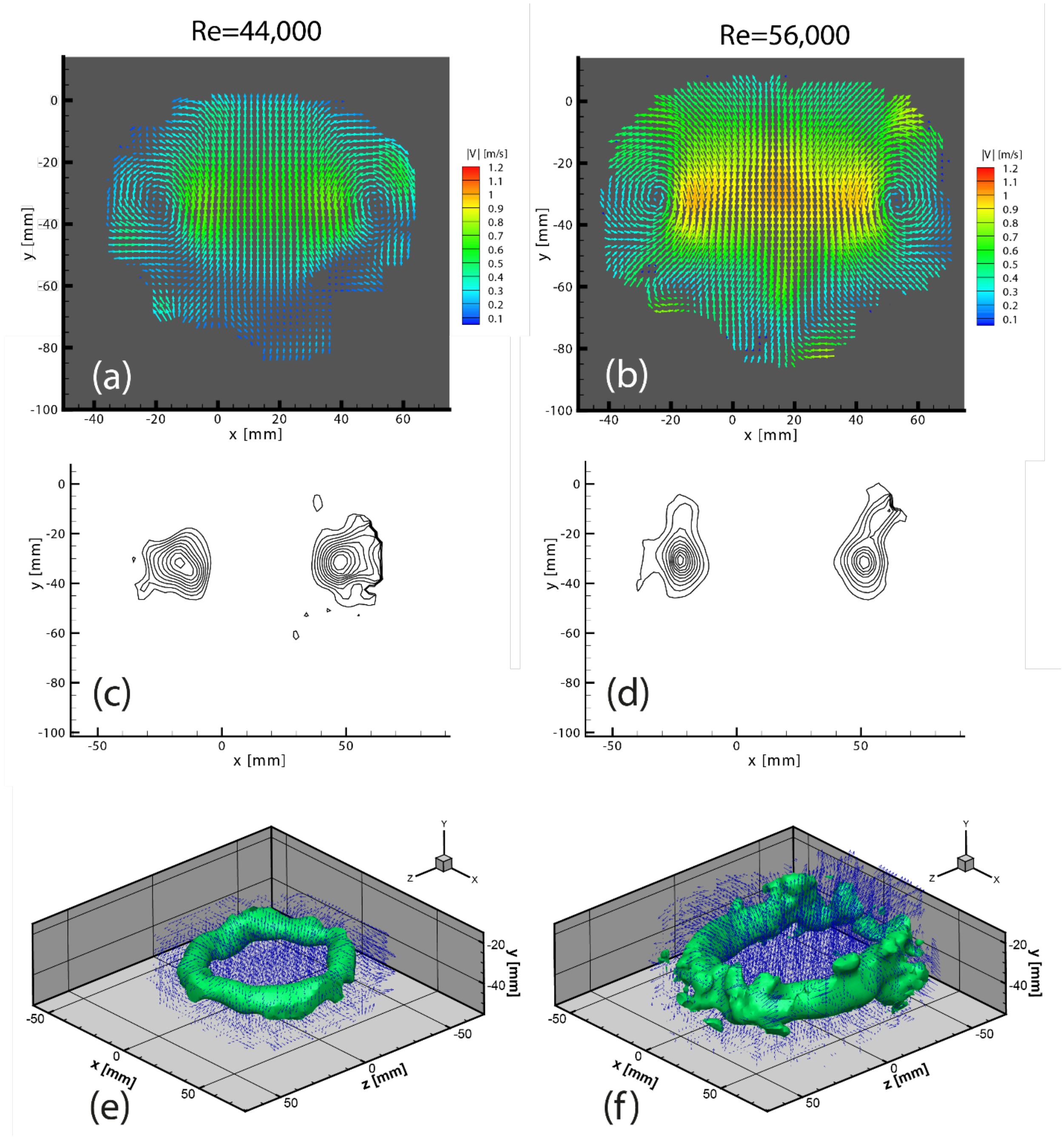

Supplementary Figure S3. Instantaneous velocity fields for a single time step in the plane z=0 mm for (a) Re=44,000, Δt=1000 μs and (b) Re=56,000, Δt=500 μs. (c,d) The corresponding iso-contours of vorticity magnitude ranging from 30 to 120 s^{-1} in (c) for the Re= 44,000 and (d) from 40 to 220 s^{-1} for Re=56,000. (e,f) The vortex ring visualized in 3D by the iso-surface of vorticity magnitude 90 s^{-1} as well as every fifth vector of the instantaneous velocity field for Re= 44,000 (e) and Re=56,000 (f).

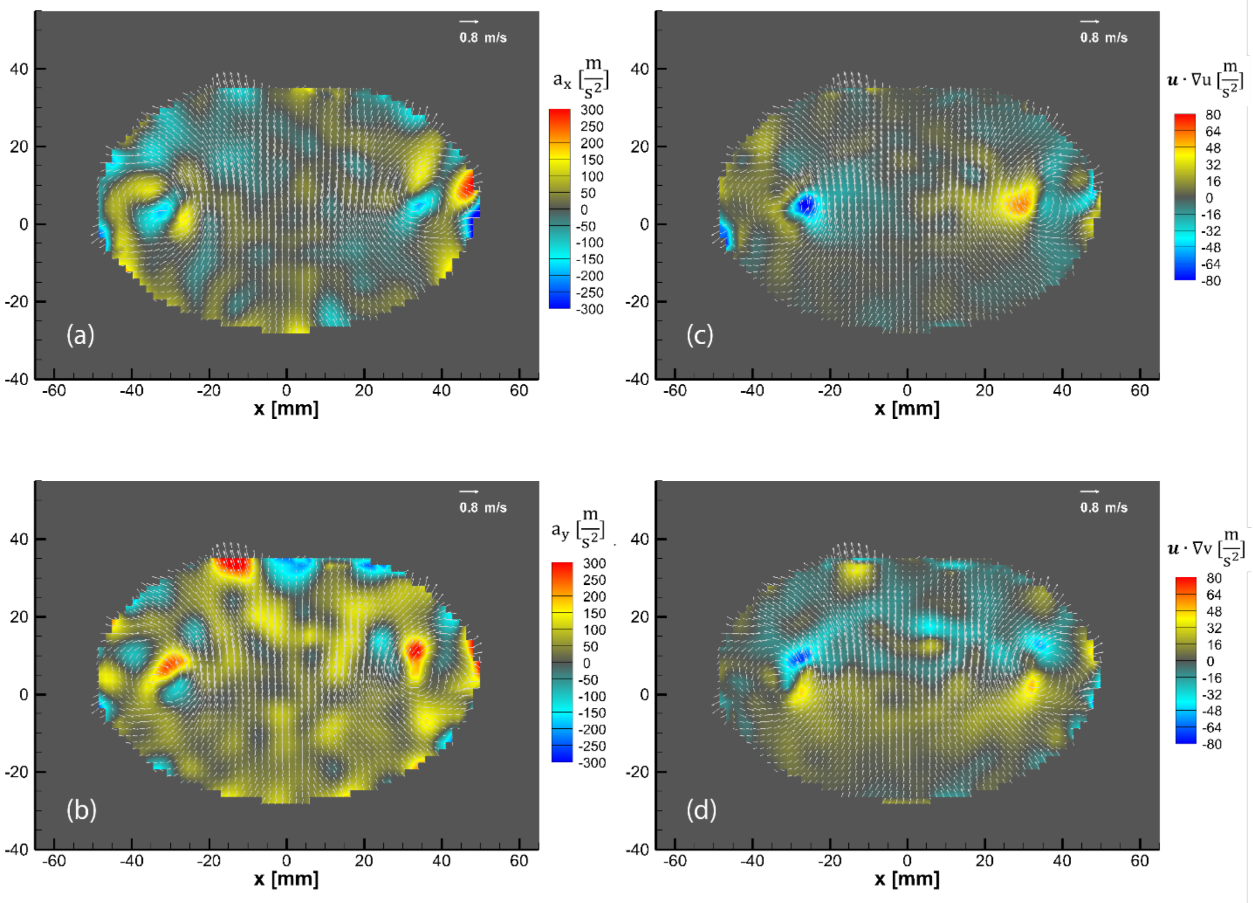

Supplementary Figure S4. Contour plots for (a,b) local acceleration ($\partial\mathbf{u}/\partial t$) and (c,d) advected acceleration (**u•∇u**) . Top figures (a,c) correspond to the X component, bottom figures (b,d) correspond to the Y component. The velocity vectors are included for position reference. The maximum local accelerations are about 4 times larger than the advected accelerations.

Circulation verification

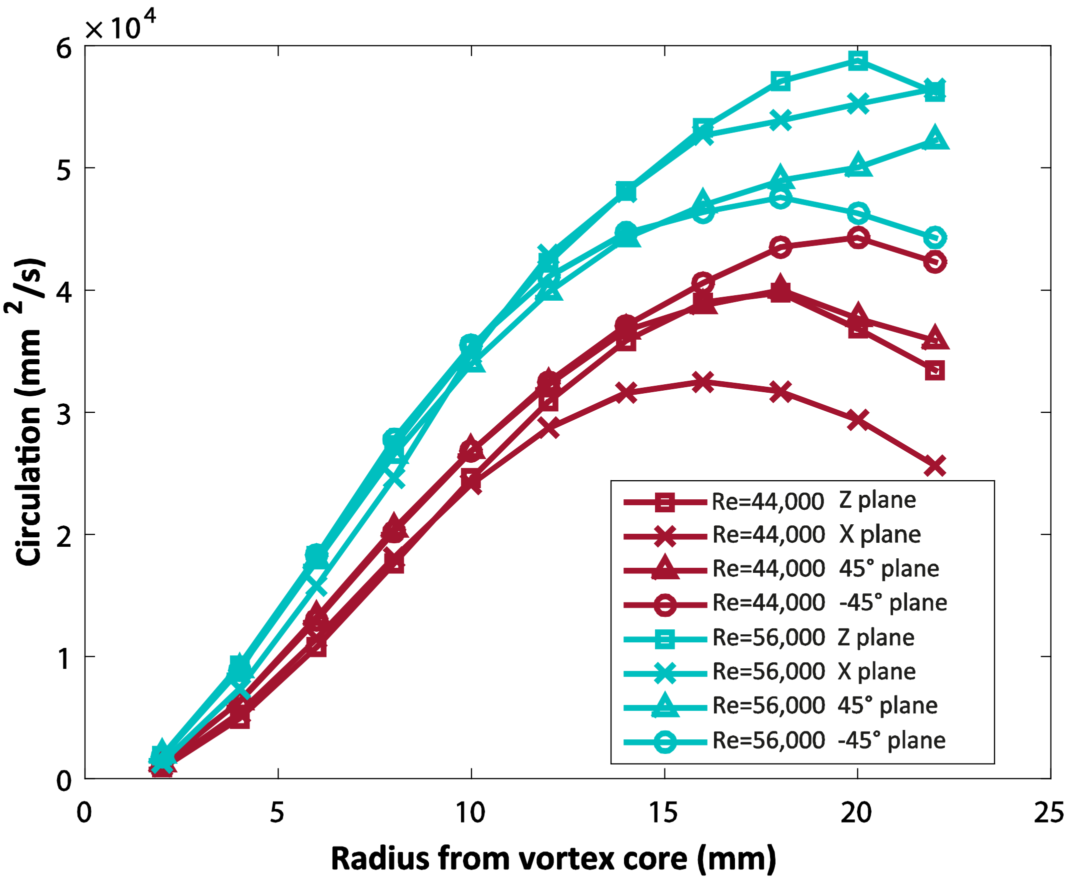

Supplementary Figure S5. Circulation Γ as a function of the radial distance from the vortex core, in four different vertical planes, for Re=44,000, Δt=1000 μs (red) and Re=56,000, Δt=500 μs (cyan). This shows that the circulation is approximately constant around every cross-section through the vortex core.

Comparison with concurrent Stereoscopic PIV measurements

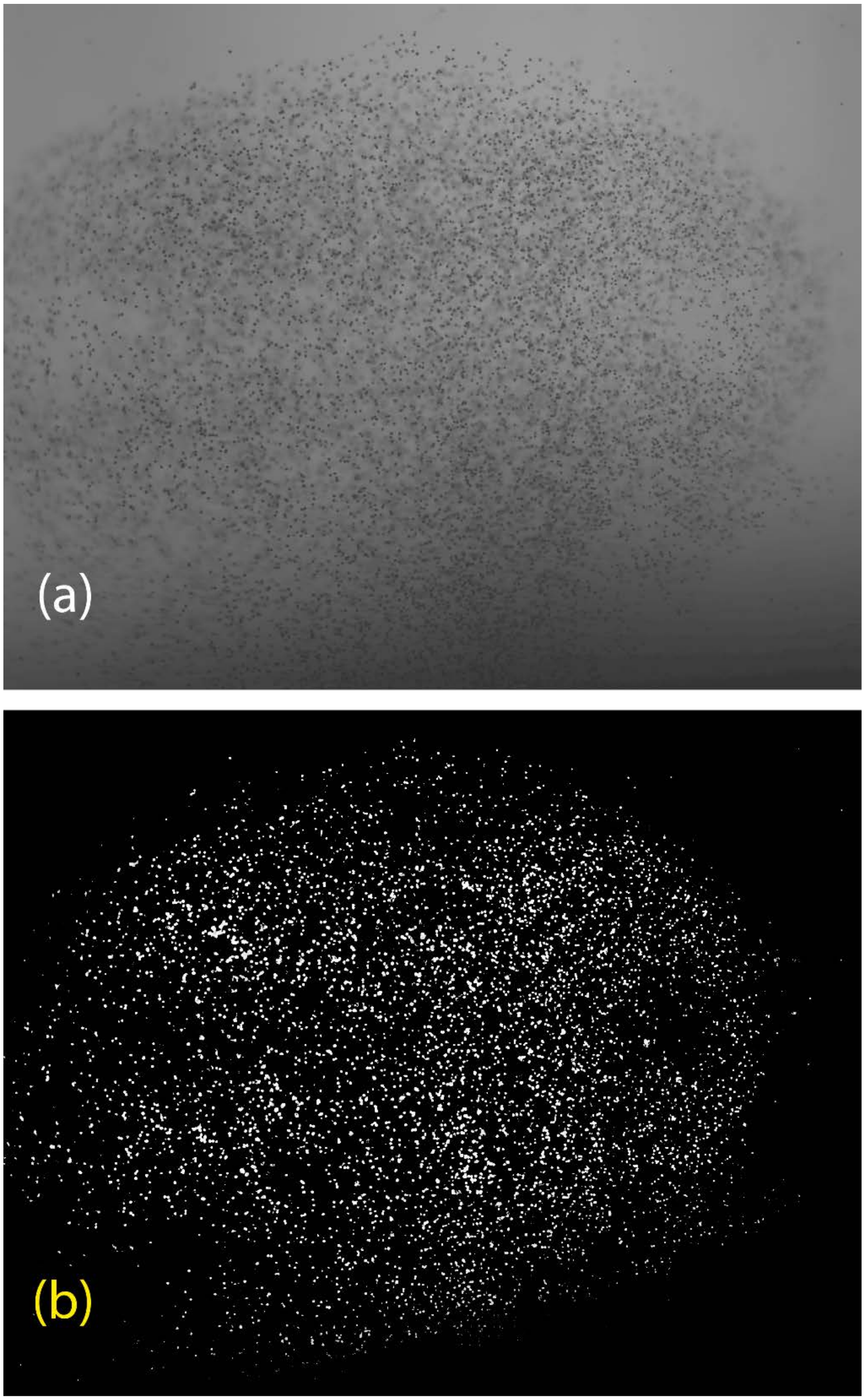

Supplementary Figure S6. (a) Original image captured by one of the CCD cameras of the stereo PIV system, notice the large background noise due to out of focus particles. (b) Particle field after image processing and filtering of the out-of-focus particle images.

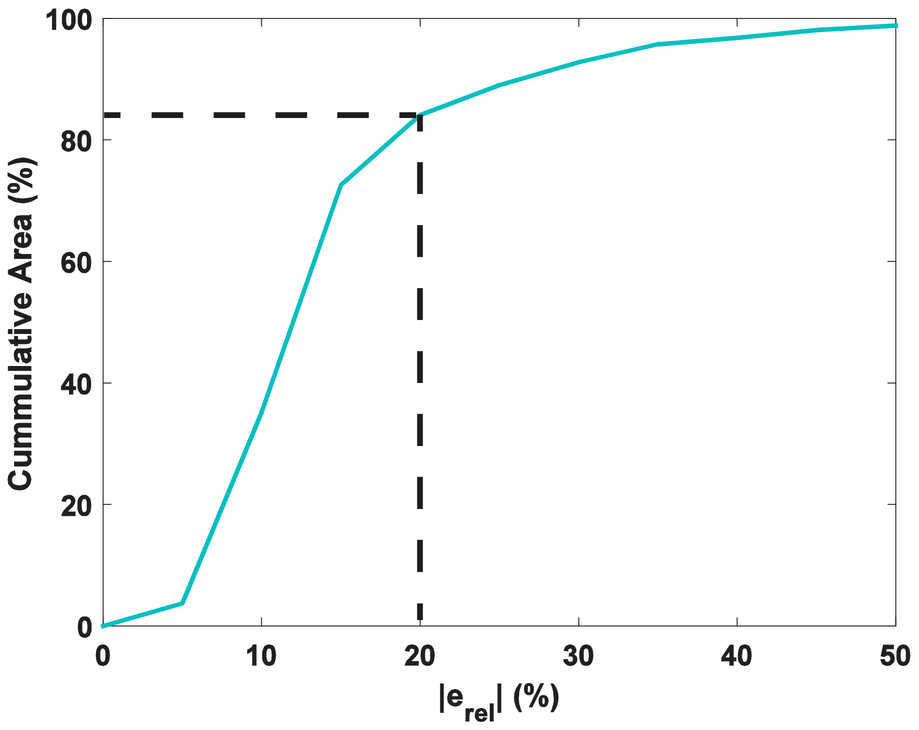

Supplementary Figure S7. Plot representing the cumulative area vs the relative error magnitude ($|e_{rel}|$) in percentage. The dashed lines clearly shows that 84% of the vectors have an error of 20% or less. Keep in mind that "error" means here the difference between the simultaneous measurements of the stereo-PIV and tomo-PIV systems.
